# Targeting the HGF/MET Axis in Cancer Therapy: Challenges in Resistance and Opportunities for Improvement

**DOI:** 10.3389/fcell.2020.00152

**Published:** 2020-05-06

**Authors:** Xing Huang, Enliang Li, Hang Shen, Xun Wang, Tianyu Tang, Xiaozhen Zhang, Jian Xu, Zengwei Tang, Chengxiang Guo, Xueli Bai, Tingbo Liang

**Affiliations:** ^1^Zhejiang Provincial Key Laboratory of Pancreatic Disease, The First Affiliated Hospital, School of Medicine, Zhejiang University, Hangzhou, China; ^2^Department of Hepatobiliary and Pancreatic Surgery, The First Affiliated Hospital, School of Medicine, Zhejiang University, Hangzhou, China; ^3^Innovation Center for the Study of Pancreatic Diseases, Zhejiang Province, Hangzhou, China

**Keywords:** hepatocyte growth factor, MET, targeted cancer therapy, therapeutic resistance, neutralizing antibody, small molecule inhibitor

## Abstract

Among hundreds of thousands of signal receptors contributing to oncogenic activation, tumorigenesis, and metastasis, the hepatocyte growth factor (HGF) receptor – also called tyrosine kinase MET – is a promising target in cancer therapy as its axis is involved in several different cancer types. It is also associated with poor outcomes and is involved in the development of therapeutic resistance. Several HGF/MET-neutralizing antibodies and MET kinase-specific small molecule inhibitors have been developed, resulting in some context-dependent progress in multiple cancer treatments. Nevertheless, the concomitant therapeutic resistance largely inhibits the translation of such targeted drug candidates into clinical application. Until now, numerous studies have been performed to understand the molecular, cellular, and upstream mechanisms that regulate HGF/MET-targeted drug resistance, further explore novel strategies to reduce the occurrence of resistance, and improve therapeutic efficacy after resistance. Intriguingly, emerging evidence has revealed that, in addition to its conventional function as an oncogene, the HGF/MET axis stands at the crossroads of tumor autophagy, immunity, and microenvironment. Based on current progress, this review summarizes the current challenges and simultaneously proposes future opportunities for HGF/MET targeting for therapeutic cancer interventions.

## Introduction

MET is a tyrosine kinase receptor with one well-established ligand, hepatocyte growth factor (HGF) (Fasolo et al., 2013). MET is expressed in several cell types, including epithelial, endothelial, neuronal, and hematopoietic cells and hepatocytes (Trusolino et al., 2010). Several human malignancies are characterized by alternating MET expression, which is usually associated with poor prognosis and aggressive phenotypes. HGF induces MET dimerization and the autophosphorylation of tyrosine residues. The subsequent recruitment of signaling effectors, including adaptor proteins Grb2, Gab-1, Src, and SHC, results in the activation of multiple downstream signaling molecules, such as mitogen-activated protein kinases (MAPK), phosphoinositide 3-kinases (PI3K), signal transducer and activator of transcription 3 (STAT3), extracellular signal-regulated kinases (ERK), phosphoinositide phospholipase C-γ, focal adhesion kinase, and nuclear factor-κB (Furge et al., 2000; Garajova et al., 2015). The activation of the HGF/MET axis is associated with a series of biological responses, such as proliferation, angiogenesis, migration, invasion, metastasis, and survival, thus contributing to the tumorigenesis, development, and progression of different human cancer types (Ghiso and Giordano, 2013). Indeed, Yamasaki et al. (2018) proved that the expression of HGF and matriptase was increased in bone metastases. Bendinelli et al. (2017) indicated the importance of targeting the tumor microenvironment by blocking epigenetic mechanisms that control critical events for colonization, such as the HGF/Met axis and WW domain-containing oxidoreductase, as a therapy for bone metastasis. The deregulation of HGF/MET signaling in tumors, including overexpression, gene amplification, activation of mutations, and increased autocrine or paracrine ligand-mediated stimulation, is caused by many different mechanisms (Migliore and Giordano, 2008).

Since the HGF/MET axis plays an important role in cancer, various approaches have been explored to inhibit it, including the use of MET-neutralizing antibodies, HGF antagonists, and tyrosine kinase activity-targeted inhibitors (TKIs). MET-specific small-molecule TKIs are divided into two functionally distinct classes: type I (e.g., crizotinib) and type II (e.g., cabozantinib) inhibitors, which preferentially bind to the active and inactive conformations of MET, respectively (Cui, 2014). Preclinical studies have demonstrated that MET inhibitors had highly efficient anti-tumor activity in some tumor types, including hepatocellular carcinoma (Bladt et al., 2014). However, several recent phase III trials using these agents failed to inhibit HGF/MET signaling (Scagliotti et al., 2015; Catenacci et al., 2017; Spigel et al., 2017). Two main factors were involved in the failure of HGF/MET-targeted drugs in clinical practice: the inappropriate selection of specific patient populations and the development of resistance to the MET-targeted drugs being administered led to poor clinical efficacy (Hughes and Siemann, 2018; Papaccio et al., 2018). The recent progress in molecular pathology and the development of more precise diagnoses may gradually solve the issue regarding the selection of specific patient populations to receive HGF/MET-targeted drugs. However, the mechanism that regulates HGF/MET-targeted drug resistance is still quite complicated. Thus, it is difficult to target HGF/MET.

It is of the utmost importance to understand the molecular mechanisms regulating resistance to specific therapies to facilitate the application of alternative agents and improve clinical efficacy through the early identification of resistance and its relevant mechanisms. Therefore, an in-depth investigation of drug resistance is critical to improving the efficacy of MET-targeted drugs. Recently, as genotype-targeted therapies have been adopted for the treatment of tumors, several new mechanisms that explain the resistance to targeted therapies have been discovered. Additionally, the resistance to MET-targeting TKIs has been partially analyzed and is mainly attributed to the mutation, fusion, and focal amplification of MET and/or other genes involved in survival. Additionally, the coordination of tumor metabolism and autophagy are critical for the resistance to HGF/MET-targeted therapy (Huang et al., 2019). Interestingly, research results have demonstrated that HGF/MET signaling is involved in the immune response (Benkhoucha et al., 2010). These studies provided new opportunities for the development of HGF/MET axis-associated basic, translational, and clinical cancer research. Therefore, this review evaluates the known mechanisms of MET resistance and highlights the recent progress in therapeutic strategies to overcome MET resistance. It also proposes future perspectives for cancer therapeutic interventions involving HGF/MET targeting.

## Multiple Mechanisms for the Resistance to Met-Targeted Therapies

### Genomic Alteration

Many studies have shown that MET point mutations and increased copy number of MET are potential mechanisms for acquired resistance to MET-TKIs (Table 1). Some studies have indicated that the Y1230 and D1228 mutations and increased MET copy number may be common mechanisms of resistance to MET inhibitors (Funakoshi et al., 2013a, b; Schrock et al., 2017). In contrast to other point mutations, the MET Y1230H mutant typically makes cells highly resistant to MET-TKIs. This MET mutation-induced resistance is irreversible (Funakoshi et al., 2013a). MET Y1230H point mutations and/or an increase in the MET copy number may result in excessive MET signaling, followed by excessive replication stress and DNA damage response, resulting in an intra-S-phase cell cycle arrest with the absence of MET-TKIs (Funakoshi et al., 2013b). A recent study identified two newly acquired MET mutations, Y1248H, and D1246N, that are resistance mechanisms for type I MET-TKIs (Li A. et al., 2017). Additionally, two studies reported mechanisms for secondary resistance to MET monoclonal antibodies: Martin et al. (2014) pointed out that MET amplification and over-expression caused resistance to MV-DN30, whereas Zhou et al. (2014) found that *p-21-activated kinase 1 (PAK1)* amplification may be the cause of resistance to onartuzumab. Moreover, onartuzumab and emibetuzumab did not achieve satisfactory clinical results in clinical trials (Shah et al., 2015; Camidge et al., 2016). Further investigation is needed to improve the clinical effect of anti-MET monoclonal antibodies and to understand the mechanisms of resistance against them.

**TABLE 1:** Link between MET alteration and therapeutic resistance.

**Targeted drugs**	**Treatment groups**	**Resistant notes**	**References**
Cabozantinib	IL-3 dependent murine pro-B cell line Ba/F3	D1133V; Y1159H; L1195F; F1200I/L	Fujino et al., 2019
Capmatinib	Lung cancer cell line EBC-1	EGFR activation; PIK3CA amplification	Ji et al., 2015
	IL-3 dependent murine pro-B cell line Ba/F3	G1090A; V1092I/L; D1164G; L1995V; M1211T; D1228A/G/H/N/Y; Y1230C/D/H/N	Fujino et al., 2019
Crizotinib	Patient with metastatic lung adenocarcinoma	Acquired EGFR mutation	Benderra et al., 2016
	Patient with NSCLC with MET exon 14 skipping	An acquired mutation in the MET kinase domain, D1228N	Heist et al., 2016
	Patient with MET exon 14-positive NSCLC	Preexisting MET Y1230C mutation	Ou et al., 2017
	Patient with lung adenocarcinomas harboring MET exon 14 splicing	D1228N/H and Y1230H mutations	Dong et al., 2016
	Mouse embryonic fibroblasts cell line NIH3T3 *in vitro* and *in vivo*	MET Y1248H and D1246N mutations	Li A. et al., 2017
	Patient with metastatic NSCLC with MET exon 14 skipping	Mutation of MET Y1230H	Schrock et al., 2017
	Patient with advanced lung adenocarcinoma with MET exon 14 skipping	MET G1163R, D1228H, D1228A, and Y1230H mutations	Zhang et al., 2017
	Patient with pulmonary adenocarcinoma harboring MET exon 14 skipping	MET exon 14 “deleting and inserting” mutation (c.3019_3028 + 29delinsACCTA, p. Phe1007fs)	Jiang et al., 2018
	Patient with ALK + NSCLC	High-level *MET* amplification	Berger et al., 2018
	Patient with NSCLC with MET exon 14 skipping	HER2 amplification	Ding et al., 2019
	Patient with advanced lung cancer with MET exon 14 skipping mutation and MET exon 5 C526F mutation	D1246N mutation	Jin et al., 2019
	IL-3 dependent murine pro-B cell line Ba/F3	V1092I/L; G1163R; D1228E/H/N/Y; Y1230C/H	Fujino et al., 2019
GSK1363089	Gastric cancer cell line MKN45	Increased copy number of *MET*	Funakoshi et al., 2013b
	Gastric cancer cell line MKN45	Increased copy number of *MET*	Funakoshi et al., 2013a
INC280	Mouse embryonic fibroblasts cell line NIH3T3	MET Y1248H and D1246N Mutations	Li A. et al., 2017
Merestinib	IL-3 dependent murine pro-B cell line Ba/F3	L1195F; F1200I/L; D1228Y	Fujino et al., 2019
MV-DN30	Lung cancer cell line EBC1	Increased MET gene copy number	Martin et al., 2014
PF-04217903	Gastric cancer cell line GTL16	SND1-BRAF fusion active MAPK pathway	Ahn et al., 2017
PHA665752	Gastric cancer cell line MKN45	Increased copy number of *MET*, and/or Y1230H mutation	Funakoshi et al., 2013b
	Gastric cancer cell line MKN45	Increased copy number of *MET*, and Y1230H mutation	Funakoshi et al., 2013a
	PDA cell line PANC-1/FG	Overexpression of FOXM1	Musiani et al., 2014
Savolitinib	PC9/Ba/F3; 293T	MET D1228V mutation	Bahcall et al., 2016
	IL-3 dependent murine pro-B cell line Ba/F3	G1090S; L1195V; D1228E; Y1230H/N	Fujino et al., 2019
Savolitinib + Osimertinib	Patient with lung adenocarcinoma	MET D1228V mutation	Bahcall et al., 2016
SGX-523	Lung cancer cell line EBC1	c-Myc alterations	Cruickshanks et al., 2019
Tepotinib	IL-3 dependent murine pro-B cell line Ba/F3	G1090S; V1155M; G1163E; D1228E/G; Y1230C/D/S/H/N	Fujino et al., 2019

*EGFR, epidermal growth factor receptor; NSCLC, non-small cell lung cancer; MAPK, mitogen-activated protein kinase; FOXM1, forkhead box M1; PIK3CA, phosphatidylinositol-4,5-bisphosphate 3-kinase, catalytic subunit alpha; ALK, anaplastic lymphoma kinase.*

Skipping mutations in MET exon 14 were reported as they are present in ∼4.0% of patients with lung cancer/lung adenocarcinoma (Baldacci et al., 2018). A non-small cell lung cancer (NSCLC) harboring a MET exon 14 skipping mutation upon treatment with crizotinib, a MET/ALK/ROS1 inhibitor, was initially reported in 2015 (Jenkins et al., 2015; Paik et al., 2015). Although crizotinib showed initial efficacy, patients inevitably acquired resistance to the drug. Several clinical reports described secondary MET mutations (D1228N/H/A; Y1230C/H) as mechanisms for crizotinib resistance (Dong et al., 2016; Heist et al., 2016; Ou et al., 2017; Schrock et al., 2017; Zhang et al., 2017). A previous study reported the case of a patient who simultaneously acquired four rare resistance mutations (G1163R, D1228H, D1228A, and Y1230H) since the development of crizotinib resistance (Zhang et al., 2017).

However, next-generation sequencing analyses have improved the detection of abnormal MET exon 14 skip mutations. Interestingly, one case of a patient with advanced lung cancer with a MET exon 14 skipping mutation and MET exon 5 C526F mutation was reported. Additionally, changes in the MET exon 14 splice site and a D1246N mutation were found during treatment with crizotinib (Jin et al., 2019). Recently, Jiang et al. (2018) reported the case of a patient with pulmonary adenocarcinoma carrying a novel MET exon 14 “deleting and inserting” mutation (c. 3019_3028 + 29delinsACCTA, p. Phe1007fs) that also led to a MET exon 14 skipping mutation, resulting in primary resistance to crizotinib. To explore the secondary resistance mechanism involving MET-TKIs, they evaluated the activity of three types of MET-TKIs using Ba/F3 cells harboring MET exon 14 mutations. They concluded that D1228 and Y1230, and L1195 and F1200 were common mutation sites that created resistance to type I and type II TKIs, respectively, as they bind to the active and inactive forms of MET, respectively. Their results indicated that tumors with resistance mutations for type I inhibitors were susceptible to type II inhibitors, and vice versa (Fujino et al., 2019). These results were consistent with those of a previous study indicating that, although the MET kinase domain mutation, D1228V, can induce tumor resistance to type I MET-TKIs through impaired drug binding, the sensitivity to type II MET-TKIs is maintained (Bahcall et al., 2016).

**FIGURE 1:**
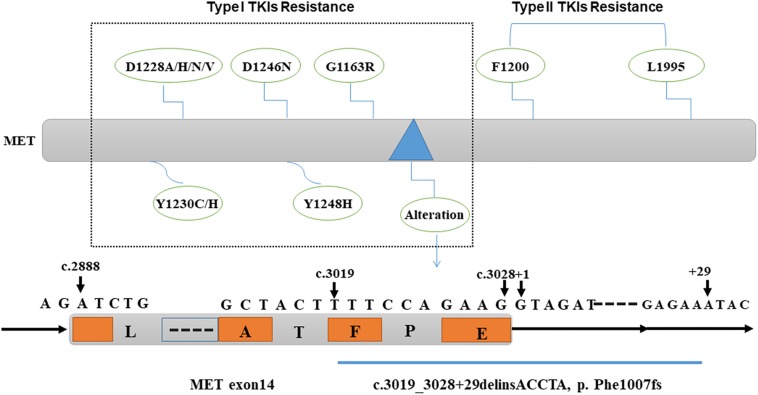
Genomic alteration causing resistance to MET-targeted therapy. MET gene mutations D1228A/H/N/V, D1246N, Y1230C/H, Y1248H, G1163R, and MET exon 14 “deleting and inserting” mutation (c.3019_3028 + 29delinsACCTA, p. Phe1007fs) cause resistance to type I MET-TKIs; MET gene mutations L1195 and F1200 are responsible for the resistance to type II MET-TKIs. TKIs, tyrosine kinase inhibitors.

Overall, MET gene alteration is an important aspect related to drug resistance against HGF/MET-targeted therapy. The detailed detection of MET gene alteration may contribute to the selection of more feasible drugs to obtain better therapeutic efficacy and clinical outcomes (Figure 1).

### Oncogenic Activation

Abnormal activation of HGF/MET signaling and its downstream pathway are present in a variety of tumors (Avan et al., 2014). In addition to point mutation and amplification of HGF/MET, the functional alteration of signaling molecules, such as the mutation of Kirsten (KRAS), Harvey rat sarcoma viral oncogene homolog (HRAS), or epidermal growth factor receptor (EGFR), and the amplification of human epidermal growth factor receptor 2 (Jan et al., 2015; Leiser et al., 2015; Benderra et al., 2016; Berger et al., 2018; Ding et al., 2019; Suzawa et al., 2019) are also involved in therapeutic resistance to HGF/MET-targeted drugs. Emerging data have suggested that MET amplification is relevant to TKI resistance in EGFR-dependent cancers, especially in lung cancer (Kwak et al., 2015; Liu et al., 2018). Meanwhile, MET inhibitors, in combination with EGFR-TKIs, may provide an effective approach to therapy for MET-positive and EGFR-TKI-resistant tumors (Engelman et al., 2007; Pasquini and Giaccone, 2018), which would be of great importance regarding the role of MET in EGFR resistance. Additionally, the activation of short-form Ron, AKT-mammalian target of rapamycin (mTOR), or the Wnt-β-catenin signaling pathway and the over-expression of STAT3, cyclooxygenase-2, c-Myc, (ATP)-binding cassette (ABC) gene 1, or heat shock protein 27 can also induce tumor resistance to MET-TKIs (Etnyre et al., 2014; Wu et al., 2014, 2015; Shen et al., 2015; Sugano et al., 2015; Cruickshanks et al., 2019). The resistance mechanisms induced by the activated HGF/MET axis pathway are summarized in Table 2.

**TABLE 2:** HGF/MET-associated oncogenic activation in therapeutic resistance.

**Targeted drugs**	**Treatment groups**	**Resistant notes**	**References**
AMG337	Patient with *MET* amplified adenocarcinoma of the distal esophagus; MET-addicted EGC cell line SNU638	*KRAS G12D* mutation	Kwak et al., 2015
	Patient with metastatic gastric adenocarcinoma with *MET* and *HER2* co-amplification	RTK co-amplification	Kwak et al., 2015
AS703026 (Pimasertib)	Gastric cancer cell line GTL-16/MKN-45; Ovarian cancer cell line CAR1/CL14	Up-regulation of HSP27	Sugano et al., 2015
AZD6244 (Selumetinib)	Gastric cancer cell line GTL-16/MKN-45; Ovarian cancer cell line CAR1/CL14	Up-regulation of HSP27	Sugano et al., 2015
Capmatinib	Lung cancer cell line EBC-1	Overexpression of EGFR-MET heterodimer	Ji et al., 2015
Crizotinib	Gastric cancer cell line GTL-16/MKN-45; Ovarian cancer cell line CAR1/CL14	Up-regulation of HSP27	Sugano et al., 2015
	Harbors concomitant amplification of *MET* and *HER2* cell line OE33, MET-addicted EGC cell line SNU638	HER2 overexpression	
	Gastric cancer cell line GTL16/SG16; Lung cancer cell line EBC-1	Deregulation of the miR-205/ERRFI1 axis and caused EGFR activation	Karagonlar et al., 2015
	Glioblastoma cell line U87/U373; Stem cell line GSC827	Upregulation of mTOR, FGFR1, EGFR STAT3, and COX-2	Etnyre et al., 2014
	Patient-derived cell line LUAD12C; H1993; NIH-3T3	*KRAS* mutation	Suzawa et al., 2019
EMD1214063	Lung carcinoma cell line H1993; NIH3T3	*KRAS* and *HRAS* mutations	Leiser et al., 2015
Emibetuzumab	Gastric cancer cell line SNU5	PTEN loss, PI3K pathway activation	Kim et al., 2019
GSK1363089	Gastric cancer cell line MKN45	Elevated the express and phosphorylation of MET, and excessive MET signaling	Funakoshi et al., 2013b
JNJ-38877605	Gastric cancer cell line GTL-16/MKN-45; Ovarian cancer cell line CAR1/CL14	Up-regulation of HSP27	Sugano et al., 2015
	Gastric cancer cell line Hs746T/MKN1	Increased HGF expression	Cui et al., 2016
	Gastric cancer cell line GTL16/SG16; Lung cancer cell line EBC-1	Deregulation of the miR-205/ERRFI1 axis and caused EGFR activation	Karagonlar et al., 2015
MV-DN30	Lung cancer cell line EBC1	Overexpression of the MET receptor	Martin et al., 2014
Onartuzumab	Pancreatic cancer cell line AsPC-1/YAPC	Dysregulation of PAK1, and PAK1 amplification	Zhou et al., 2014
	Glioblastoma cell line U87/U373; Stem cell line GSC827	Upregulation of mTOR, FGFR1, EGFR, STAT3, and COX-2	Etnyre et al., 2014
PD98059	Gastric cancer cell line GTL-16/MKN-45; Ovarian cancer cell line CAR1/CL14	Up-regulation of HSP27	Sugano et al., 2015
PHA665752	Gastric cancer cell line MKN45	Elevated the express and phosphorylation of MET, and excessive MET signaling	Funakoshi et al., 2013b
	Gastric cancer cell line GTL-16	Truncated RAF1 and BRAF proteins	Lee et al., 2012
	Lung cancer cell line EBC-1	Activation of KRAS, and activation of EGFR and FGFR2 signaling by a MET-independent bypass pathway; miR-138 regulate ABCB1 overexpression	Shen et al., 2015
	Gastric cancer cell line Hs746T/MKN1	Increased HGF expression	Cui et al., 2016
	Gastric cancer cell line GTL16/SG16; Lung cancer cell line EBC-1	Deregulation of the miR-205/ERRFI1 axis and caused EGFR activation	Karagonlar et al., 2015
Savolitinib	Lung cancer cell line H1993/EBC-1	Aberrant mTOR activation; MYC over-expression; Activation of EGFR signaling; and PIM kinases	An et al., 2015
SU11274	Melanoma cell line MU/RU	Activation of Akt/mTOR and Wnt/β-catenin pathways	Wu et al., 2015

*EGFR, epidermal growth factor receptor; PIK3; phosphatidylinositol-4,5 -bisphosphate 3-kinase; KRAS, Kirsten rat sarcoma viral oncogene homolog; HRAS, Harvey rat sarcoma viral oncogene homolog; PAK1; p-21-activated kinase 1; FGFR, fibroblast growth factor receptors; COX-2; cyclooxygenase-2; STAT3, signal transducer and activator of transcription 3; HGF, hepatocyte growth factor; mTOR, mammalian target of rapamycin; MYC, myelocytomatosis viral oncogene; RTK, receptor tyrosine kinase; PIM, proviral integration site for Moloney murine leukemia virus; PTEN, prime time entertainment network; ABCB1, adenosine triphosphate (ATP)-binding cassette; ERRFI1, ERBB receptor feedback inhibitor-1; HSP, heat shock protein; HER2, human epidermal growth factor receptor 2; miR; micro-RNA.*

HGF/MET signaling is aberrantly activated in different solid tumors and associated with poor prognosis (Avan et al., 2014). Cui et al. (2016) found that the activation of HGF/MET signaling increases the expression and transcriptional activity of forkhead box protein M1 (FOXM1) through ERK, PI3K, and STAT3. Moreover, FOXM1 can bind to the *MET* gene promoter to increase the expression of MET at the transcriptional level. The positive feedback between HGF/MET and FOXM1 signaling promotes the growth of pancreatic ductal adenocarcinoma and induces resistance to MET inhibition (Musiani et al., 2014). Specifically, HGF overexpression leads to MET-TKI resistance through an autocrine mechanism in gastric cancer cells (Cui et al., 2016). The activated SND1-BRAF fusion protein, caused by an amplified chromosomal rearrangement between 7q32 and 7q34, contains a constitutively active BRAF kinase that increases ERK phosphorylation and consequent hyperactivation of the downstream MAPK pathway, eventually leading to resistance to MET-TKI (Ahn et al., 2017). A similar outcome has been observed in another study, where truncated RAF1 and BRAF were identified as significant determinants of the resistance to MET inhibition in GTL-16 cells (Lee et al., 2012).

Some studies have demonstrated that the HGF/MET axis-activated downstream PI3K signaling pathway plays an important role in tumor resistance to MET inhibitors. For instance, Ji et al. (2015) demonstrated that the MET-addicted SNU-5 xenograft model developed resistance to MET inhibitors due to PI3K p110α gene overexpression. A combination of the two inhibitors, PHA665752 and PI-103, exerts a significant synergistic anti-tumor effect on PHA665752-resistant xenografts *in vivo* (Petti et al., 2015). Recently, Kim et al. (2019) showed that increased MET and EGFR hetero-dimerization could result in acquired resistance to capmatinib. Their study indicated that the activation of EGFR signaling and/or genetic alteration of the downstream effector phosphatidylinositol-4,5-bisphosphate 3-kinase catalytic subunit alpha (PIK3CA) are alternative resistance mechanisms used by capmatinib-resistant NSCLC cell lines. Hence, a combined treatment of MET, EGFR, and PI3K inhibitors may be an effective therapeutic strategy in patients with capmatinib-resistant NSCLC (Ji et al., 2015). Moreover, dysfunction of the PI3K pathway is linked to resistance to anti-MET antibodies. Especially, Pollmann et al. (2018) identified two potential mechanisms of resistance, both involving PI3K pathway activation, according to their long-term *in vivo* models of either acquired resistance to the MET-targeting antibody emibetuzumab due to PTEN loss or increased receptor tyrosine kinase activation through increased MYC and ERBB3 copy numbers. Furthermore, Sym015, a mixture of two monoclonal antibodies that bind to non-overlapping MET epitopes, effectively prevents or reduces these resistances due to its broader mechanism of action (Kim et al., 2019).

Additionally, a few studies have shown that the kinase activity of the proviral integration site for Moloney murine leukemia virus (PIM) is required to acquire resistance to MET inhibitors in the MET-dependent tumor model. PIM 1/3 upregulation is associated with acquired resistance to MET inhibitors. PIM kinases mediate resistance to MET inhibitors through the control of cap-independent Bcl-2 translation (Pollmann et al., 2018). Indeed, Henry et al. (2016) demonstrated that resistance to savolitinib (a small-molecule inhibitor of MET) could be mediated by PIM kinase signaling, and they showed PIM inhibition restores savolitinib sensitivity *in vitro* and *in vivo* (An et al., 2015).

A number of publications have reported that several micro-RNAs (miRNAs) inhibit tumor progression by targeting MET (Zheng et al., 2015; Henry et al., 2016). Furthermore, the role of miRNAs in MET-TKI resistance has been confirmed in preclinical models. Specifically, Migliore et al. (2018) showed that miR-205 upregulation is associated with the resistance of MET-addicted tumors to structurally different MET-TKIs (non-selective, such as crizotinib, or selective, such as PHA-665752 and JNJ-38877605) via ERRFI1 targeting and consequent EGFR activation (Karagonlar et al., 2015).

**FIGURE 2:**
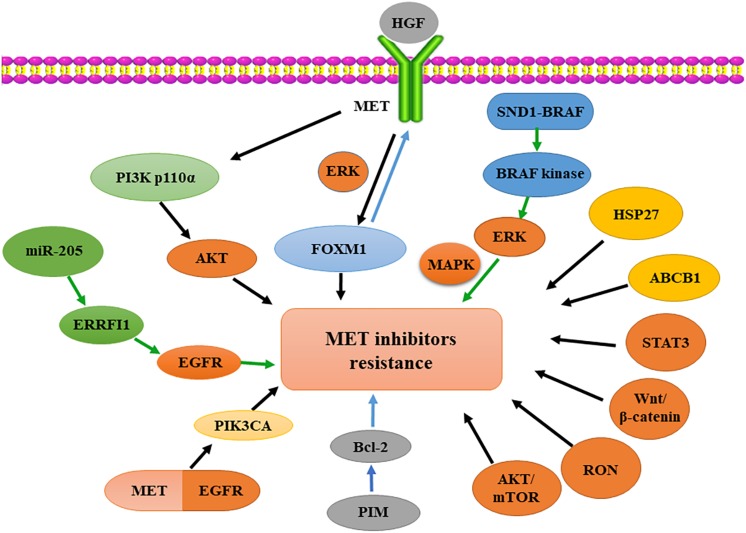
Oncogenic activation involved in resistance to MET-targeted therapy. Activated MET regulates FOXM1 expression in therapeutic resistance via ERK signaling. SND1-BRAF activates BRAF kinase, subsequently phosphorylating ERK and activating MAPK. Overexpression of HSP27 or ABCB1 and activation of STAT3, Wnt/β-catenin, RON or AKT/mTOR signaling pathways induce resistance to MET inhibition. PIM kinase regulates Bcl-2 translation in resistance to MET inhibition. MET-EGFR heterodimerization activates PIK3CA; PI3K p110α overexpression induces resistance to MET inhibition by activated AKT. MiR-205 upregulation reduces the expression of ERRFI1 and increases EGFR activity, causing MET-targeted resistance. HGF, hepatocyte growth factor; FOXM1, forkhead box M1; ERK, extracellular signal-regulated kinase; MAPK, mitogen-activated protein kinase; HSP27, heat-shock protein 27; ABCB1, ATP-binding cassette subfamily B member 1; STAT3, signal transducers and activators of transcription 3; RON, receptor originated from Nantes; mTOR, mammalian target of rapamycin; AKT, protein kinase B; EGFR, epidermal growth factor receptor; PI3K, phosphatidylinositide 3-kinase; ERRFI1, ERBB receptor feedback inhibitor-1; PIK3CA, phosphatidylinositol-4,5-bisphosphate 3-kinase, catalytic subunit alpha; PIM, proviral integration site for Moloney murine leukemia virus; miR, micro-RNA.

Overall, acquired resistance to HGF/MET-targeted therapy can occur due to a variety of mechanisms: (i) second-site mutations in the MET kinase domain or abnormal activation of the HGF/MET signaling pathway, (ii) oncogene overexpression or the induction of bypass signaling pathways, (iii) copy number changes, and (iv) upregulation of downstream signaling molecules or formation of fusion proteins (Sequist et al., 2011; Ohashi et al., 2013; Migliore et al., 2018). Therefore, a major priority of researchers and physicians is to identify the underlying mechanisms of MET-TKI resistance in each patient through molecular studies to establish a more effective therapeutic strategy for the management of drug resistance (Figure 2).

### Autophagic Compensation

Autophagy, one of several cellular adaptive responses to therapeutic stresses caused by anti-cancer agents, is an evolutionarily conserved proteolytic process in which damaged cellular components are incorporated into lysosomes for degradation and material recycling (Lin et al., 2017). Thus, autophagy inhibition has been used concurrently with chemotherapies or targeted therapies to optimize their efficacy in preclinical studies on various cancer types (White, 2015).

A complicated and diverse link exists between the HGF/MET signaling pathway and cellular autophagy (Gallo et al., 2014; Shen et al., 2017). For instance, Maroni et al. (2014) utilized adenovirally expressed NK4 (AdNK4) plus dasatinib (DAS) inhibitor to block the HGF/MET axis and Src activity, and the combination of AdNK4 and DAS resulted in a state of autophagy failure state, marked by p62 without Beclin 1, that largely prevented osteolytic bone metastases through the dysfunctional interplay between autophagy and anoikis (Barrow-McGee et al., 2016). Li N. et al. (2017) found that the *Helicobacter pylori* CagA protein negatively regulates autophagy via the MET-PI3K/AKT-mTOR signaling pathway (Maroni et al., 2014). Meanwhile, they also demonstrated that autophagy enhances the chemosensitivity of papillary thyroid cancer by inhibiting MET. Their study demonstrated that RAD001, an anti-tumor agent, induces an autophagy-related protein expression that increases autophagy, eventually leading to MET de-phosphorylation, and consequently enhances the chemotherapeutic response (Li N. et al., 2017). Increasing evidence shows that resistance to MET-targeted therapy is closely related to tumor cell autophagy. Our most recent study confirmed that HGF/MET kinase-targeted drugs promoted autophagy, and the inhibition of autophagy enhances the killing effects of MET-targeted drugs on human and murine liver cancer cells. We further found that the key residues for MET kinase activity (Y1234/1235) represent a conserved LC3-interacting region motif (Y1234-Y1235-x-V1237). Furthermore, we found that Y1234/1235-dephosphorylated MET is closely related to the autophagic state found in human liver cancer specimens, and a combination of MET and autophagy inhibitor significantly improved the therapeutic effect against liver cancer (Lin et al., 2010). Previous studies further showed that the MET inhibitors EMD1214063 and PHA665752 promote protective autophagy and cause tumor cell resistance to human gastric cancer cells (Humbert et al., 2013). This is consistent with the conclusion proposed by Lin et al., which stated that MET inhibitors could induce autophagy-related protein expression and flux increase. MET inhibitors increase autophagy in gastric cancer cells through the mTOR and ULK1 de-phosphorylation mechanisms. The combined use of MET and autophagy inhibitors can effectively inhibit tumor growth (Lin et al., 2019).

Although the aforementioned studies suggest that MET inhibitors can induce protective autophagy in tumor cells, which allows these cells to acquire resistance to MET inhibition, it is yet not clear whether a combination of inhibitors targeting both MET and autophagy could improve the therapeutic efficacy. Schroeder et al. (2017) found that autophagy inhibitors are not effective at enhancing the efficacy of MET inhibitors. Their study showed that crizotinib is sufficient to promote autophagy, and autophagy inhibitors prevent cytochrome c release, thus attenuating apoptosis. Therefore, they speculated that autophagy is required for crizotinib-induced apoptosis in MET-amplified gastric cancer cells (Schroeder et al., 2017). Similarly, one study reported that MET inhibitors increased the ratio of autophagy and the inhibition of autophagy down-regulated oridonin-induced apoptosis. They also found that apoptosis increases autophagy in cells co-treated with oridonin and SU11274 (a MET inhibitor) (Liu et al., 2013).

**FIGURE 3:**
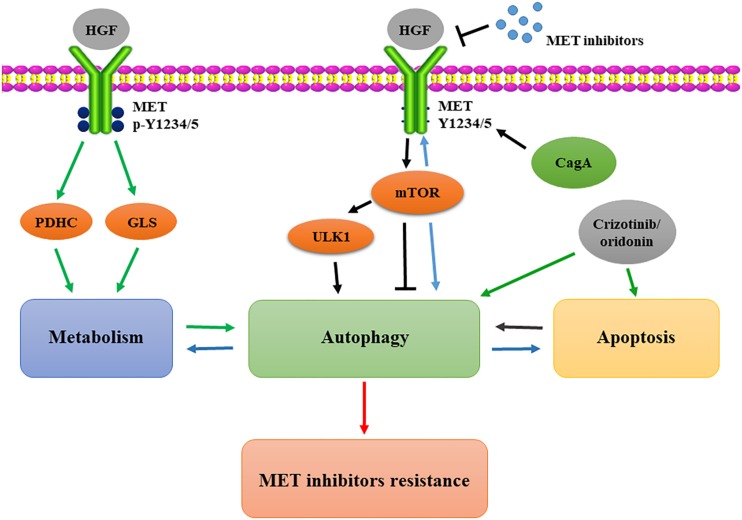
Autophagic compensation in resistance to MET-targeted therapy. CagA protein negatively regulates autophagy via the MET-mTOR signaling pathway; MET inhibition induces mTOR activation and ULK1 dephosphorylation to increase autophagy; Y1234/1235 dephosphorylation of MET mediates the metabolic transformation to autophagy; crizotinib and oridonin strengthen the positive feedback loop between autophagy and apoptosis in the resistance. mTOR, mammalian target of rapamycin; PDHC, pyruvate dehydrogenase complex; GLS, glutaminase; ULK1, UNC-51–like kinase.

Taken together, the aforementioned reports highlight that, despite the fact that MET inhibitors effectively promote autophagy, this process is still dependent on the specific biological conditions of the cells, such as the stress to which they are subjected. Therefore, the mechanism of autophagy-induced MET inhibitor resistance needs further confirmation (Figure 3).

### Immunological Regulation

Accumulating evidence suggests a close relationship between MET and immune regulation. For instance, constitutive MET expression was observed in hematopoietic progenitor cells and antigen-presenting cells, including B cells, monocytes/macrophages, and dendritic cells (DCs). Moreover, exposure to pro-inflammatory cytokines can lead to the induction or upregulation of MET expression in various cell types. In addition, HGF/MET signaling affects numerous immune cells, including mast cells (McCall-Culbreath et al., 2008), DCs (Baek et al., 2012), Langerhans cells (Sagi and Hieronymus, 2018), and neutrophils (Finisguerra et al., 2015).

The binding of *Listeria monocytogenes* internalin B to MET provides the functionality required for peritoneal mast cell activation, and crosstalk between MET and α2β1 integrin may contribute to mast-cell activation in autoimmune conditions (McCall-Culbreath et al., 2008). Baek et al. (2012) found MET signaling regulated matrix metalloproteinase (MMP)2 and MMP9 activity, and unveiled MET signaling in DCs as a critical determinant for the maintenance of normal immune function. When the HGF/MET signal was blocked in DCs, dysfunction in the migration and activation of innate immune cells was observed (Baek et al., 2012). HGF was shown to impair DC activation, resulting in a diminished antigen-presenting capacity (Okunishi et al., 2005; Singhal and Sen, 2011). Furthermore, HGF-treated DCs showed increased expression of programmed cell death 1 ligand 1 (PD-1 ligand 1, PD-L1) and IL-27 (Benkhoucha et al., 2014). However, in response to infection or tissue injury, the production of HGF is further stimulated by pro-inflammatory cytokines, whereas anti-inflammatory factors inhibit HGF production (Gohda et al., 1992a, b; Inaba et al., 1993; Tamura et al., 1993; Liu et al., 1994). Therefore, our understanding of HGF/MET signaling mechanisms is still in its infancy and should be extended.

MET is required for chemoattraction and cytotoxicity of neutrophil in response to its ligand, HGF. MET deletion in neutrophils leads to the enhancement of tumor growth and metastasis (Finisguerra et al., 2015). Thus, MET blockade in antitumor neutrophils limits the therapeutic efficacy of systemic MET inhibitors (No authors listed, 2015). However, Glodde et al. (2017) found that in the absence of MET inhibition, neutrophils were recruited to T cell-inflamed microenvironments (with CD8 + T cell infiltration and type I interferons) (Woo et al., 2015; Spranger, 2016) and rapidly acquired immunosuppressive properties, thus restraining T cell expansion and effector function. This result demonstrates a role for the HGF/MET pathway in neutrophil recruitment and function and suggests that MET inhibitor co-treatment may improve responses to cancer immunotherapy in MET-independent tumors by directly activating T cell-mediated anti-cancer immunity (Glodde et al., 2017). Overall, MET inhibitors affect MET expression in tumor-associated immune cells and are a potential mechanism for drug resistance.

**FIGURE 4:**
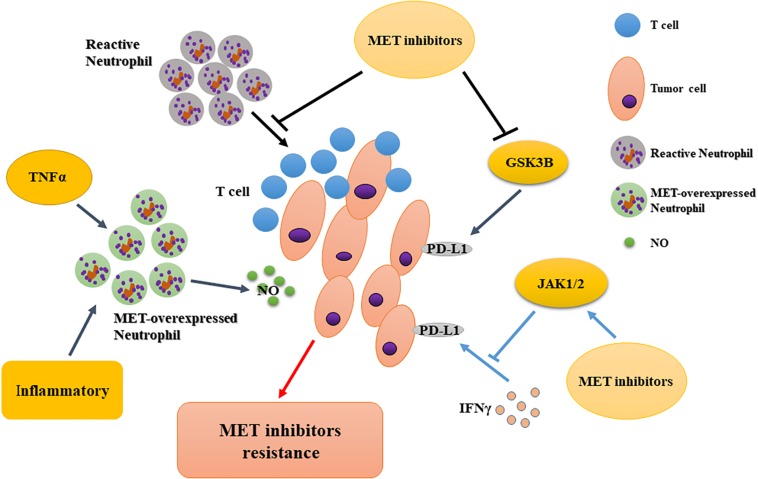
Immunological regulation of resistance to MET-targeted therapy. MET inhibition impairs the recruitment of reactive neutrophils to tumor cells, potentiating T cell-mediated anti-tumor immunity; tumor-derived TNFα or inflammatory stimuli increase MET expression in anti-tumor neutrophils to release more nitric oxide, facilitating tumor cell killing; MET inhibitors downregulate PD-L1 expression via different signaling molecules. PD-L1, programmed cell-death ligand 1; GSK3B, glycogen synthase kinase 3β; JAK, Janus kinase; TNFα; tumor necrosis factor α.

Many recent studies have focused on the application of immune checkpoint inhibitors in combination with other drugs. Li et al. showed that MET downregulates PD-L1 expression through the phosphorylation and activation of glycogen synthase kinase 3β. Experiments confirmed that MET-specific inhibitors upregulate the expression of PD-L1, thereby compromising the tumor-killing effect of MET inhibitors (Li et al., 2019). However, Martin et al. discovered that PD-L1 and PD-L2 were upregulated in MET-amplified tumor cells after interferon-γ (IFNγ) treatment. MET inhibitors could neutralize the activation of Janus kinases/STAT1 (signal transducers downstream of the IFNγ receptor) and counteract the induction of PD-1 ligands by IFNγ in MET-amplified cancers (Martin et al., 2019). Interestingly, both articles concluded that MET inhibitors combined with immune checkpoint inhibitors might have additional clinical benefits. Therefore, the mechanisms of resistance to MET inhibitors should be confirmed by further research involving immune checkpoints. The context-dependent effects of MET on PD-L1 and other immune checkpoints may be a promising research direction to overcome HGF/MET-related drug resistance (Figure 4).

### Microenvironmental Interference

The tumor microenvironment includes fibroblasts, the extracellular matrix (ECM), immune and other cells, and molecules. It has recently been recognized as an important factor in sustained resistance to targeted therapies. For example, the production of ligands in a paracrine manner can activate signals that are able to compensate for drug-inhibited pathways in tumor cells (Harbinski et al., 2012; Straussman et al., 2012; Wilson et al., 2012). Tumor-associated fibroblasts are important factors in the tumor microenvironment and constitute a heterogenous group of fibroblasts whose function is pirated by cancer cells and redirected toward carcinogenesis (Marsh et al., 2013). Tumor-associated fibroblasts secrete HGF, whose overexpression activates downstream signals, causing primary or secondary tumor resistance (Straussman et al., 2012; Wilson et al., 2012).

Ahn et al. (2017) also showed that HGF caused MET inhibitor resistance in a paracrine manner. MET inhibitors inhibit proliferation, invasion, migration, and HGF/MET downstream signaling in gastric MET-amplified cancer cells, but HGF overexpression in cancer cells impairs this phenomenon. Additionally, HGF promotes the formation of anchorage-independent colonies of MET-amplified gastric cancer cells (Cui et al., 2016). Apicella et al. (2018) showed that lactate enhanced the formation of HGF by tumor-associated fibroblasts, which in turn activated MET-dependent signaling pathways in cancer cells, causing resistance to MET inhibitors. Tumor-associated fibroblasts are able to rework the ECM to include more paracrine HGF. HGF overexpression further affects the activation of MET and its downstream signaling pathways, thereby causing tumor resistance to MET inhibitors.

**FIGURE 5:**
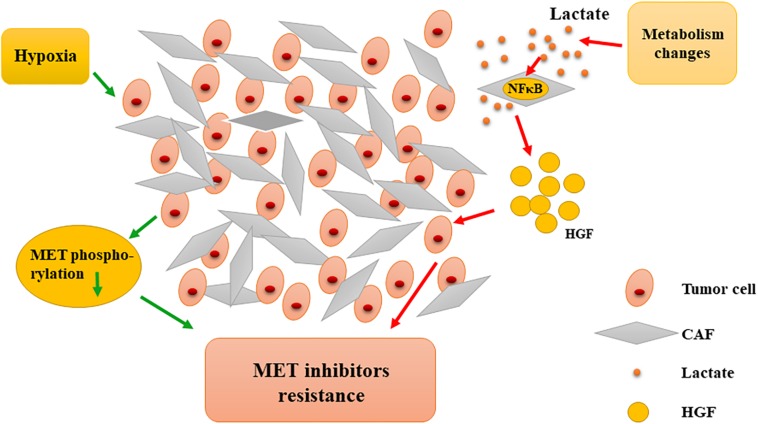
Microenvironmental interference in resistance to MET-targeted therapy. Hypoxia induces the reduction of MET phosphorylation, causing resistance to MET inhibitors. Metabolic changes contribute to tumor cells’ resistance to MET inhibitors by promoting CAFs to secrete more HGF. CAF, cancer-associated fibroblast; HGF, hepatocyte growth factor; NFκB, nuclear factor-κB.

In addition, hypoxia is a characteristic of the tumor microenvironment and is closely related to resistance to MET-targeted inhibitors. Recent studies have shown that under hypoxic conditions, tumor cells developed resistance to MET inhibitors (PHA-665752 and SU11274) and, interestingly, could recover their sensitivity to MET-TKI when normoxia was restored. Hypoxia significantly reduces the phosphorylation of critical MET residues, whereas MET downstream signaling, AKT, and ERK are not affected. After restoration from hypoxia, MET phosphorylation and activity can be quickly recovered (Mekki et al., 2018). These phenotypes indicate that there are functional transfers between MET and other similar factors when switching from hypoxia to normoxia and vice versa. Moreover, MET-TKI inhibitor resistance under hypoxia may be partially dependent on the sustained activation of the HGF/MET downstream signaling pathway. As previously mentioned, alteration of MET phosphorylation may also cause tumor cell resistance. Hypoxia is caused by a long-term lack of blood supply and is seen in a variety of solid tumors. Thus, the failure of MET inhibitors in multiple clinical phase III trials was not surprising. Therefore, the microenvironment-relevant mechanisms for resistance to MET inhibition should be further analyzed (Figure 5).

## Recent Advances in the Improvement of Met-Targeted Therapeutic Efficacy

Although many inhibitors and antibodies against HGF/MET are available, most of them are designed to block HGF-mediated MET activation or directly inhibit the kinase activity of MET. However, due to the aforementioned reasons, the targeting mechanisms of MET inhibitors may potentially cause drug resistance and poor clinical efficacy. Therefore, it is important to improve the clinical efficacy of MET inhibitors to reduce the occurrence of drug resistance. According to these needs, we have constructed an anti-MET single-domain nanoantibody (VHH) pool to improve the efficacy of MET inhibitors. The anti-MET VHH pool against the whole MET ectodomain can effectively decrease MET phosphorylation and protein expression, and inhibit tumor cell proliferation, invasion, and tumor growth (Su et al., 2019). However, further studies are still needed to examine its potential clinical effects and establish its suitability for patients with MET-inhibitor resistance. In addition, recent studies suggest that the MET antibody mixture, Sym015, overcomes acquired resistance. An emibetuzumab-resistant cell line remained sensitive to the Sym015 antibody, which can induce antibody-dependent cell-mediated cytotoxicity to inhibit tumor growth, thereby overcoming emibetuzumab resistance (Kim et al., 2019).

As we have previously explained, the HGF/MET signaling pathway is closely related to tumor immunity and, thus, researchers reconsidered an HGF/MET-targeted strategy for immunological stimulation and activation to obtain better tumor-killing effects. A bispecific monoclonal antibody targeting MET and PD-1 has been designed, developed, and tested for multiple cancer models by Sun et al. (2017). They achieved good preclinical results, revealing that the bispecific MET/PD-1 antibody could effectively promote the degradation of MET protein. Thus, the antibody inhibits the activation of HGF/MET axis downstream signaling and tumor growth, and reduces the release of the inflammatory factor IL-6, thus suggesting a vast therapeutic potential (Sun et al., 2017). Similarly, Thayaparan et al. (2017) reported that NK1-targeted chimeric antigen receptors mediated MET-dependent T-cell activation and the destruction of mesothelioma cells. Since the HGF/MET signaling pathway plays a crucial role in tumor progression, the design of new antibodies and inhibitors considering different perspectives is of great value for improving the efficacy of MET-targeted drugs. MET inhibitors or antibody drugs resulted in excellent results in preclinical studies, but no satisfactory clinical outcomes have been obtained in subsequent clinical studies. Therefore, improvements in the efficacy of MET inhibitors should be further explored in future studies.

## Further Considerations

Acquired resistance to targeted therapy can occur through numerous mechanisms, including MET point mutations, increasing copy number, and bypassing signaling pathway activation. Some studies suggested that switching from type I to type II inhibitors, or vice versa, results in an acquired resistance mutation to each type (Engstrom et al., 2017; Reungwetwattana et al., 2017). For instance, Engstrom et al. (2017) reported that MET-addicted SNU-638 gastric cancer cells with D1228N and Y1230C/H MET mutations were resistant to type I MET-TKIs but sensitive to glesatinib (type II) (Engstrom et al., 2017). However, switching from type I to type II, or vice versa, may not always be effective in MET-TKI resistant tumors. Some secondary mutations, such as D1228A/Y, cause tumor resistance to both type I and type II MET-TKIs. Moreover, concurrent aberrations, such as KRAS mutation or amplification with MET amplification and the upregulation of HGF, may also have an influence on sensitivity to MET-TKIs (Bahcall et al., 2018; Suzawa et al., 2019). Additionally, unsatisfactory outcomes of the clinical applications of drugs targeting MET kinase activity or activation suggest the need to consider whether the kinase-independent functions of MET have been neglected. For instance, MET is closely related to tumor autophagy, metabolism, and microenvironment, all of which are not entirely dependent on the kinase activity of MET.

Cancer promotion and survival depend on the complex signaling network between tumor and stromal cells in the surrounding microenvironment. Furthermore, HGF is secreted by both cancer and stromal cells. As Katayama et al. (2012) suggested, the stromal secretion of stem cell factors leads to the activation of its receptor, KIT, which may promote crizotinib resistance. Likewise, Apicella et al. (2018) reported that cancer-associated fibroblasts produce HGF, further activating MET-dependent signaling in cancer cells and resulting in sustained resistance to TKIs. Therefore, the paracrine factors of tumor and stromal cells have a potentially important role in driving TKI resistance.

The HGF/MET pathway is considered a promising target in multiple cancer types. Resistance is the result of the complex interactions among various receptor tyrosine kinases and other proliferative signals. Thus, the mere inhibition of MET phosphorylation with a single drug treatment may be insufficient to suppress the HGF/MET pathway (Morgillo et al., 2017). Encouraging preclinical results have shown that the combination of MET-TKI and immunotherapy achieved effective anti-tumor effects and also brought new hope for the next-generation of MET-TKIs.

Taken together, the role of HGF/MET in cancer seems to be quite complicated. HGF/MET has oncogenic and pro-metastatic effects, but also has anti-cancer functions. Accumulating evidence suggests a close relationship between the HGF/MET signaling pathway and induction of the immune response. Thus, it remains uncertain whether it acts as an immunosuppressive or an immune-positive stimulus. Therefore, more in-depth investigations of these controversies should be performed to provide a better understanding of the mechanisms of resistance and accelerate the development of next-generation MET-TKI-targeting drugs.

## Author Contributions

XH, XB, and TL designed this project and supervised the work. XH, EL, and HS performed the reference collection, analysis, and interpretation, wrote the manuscript, and contributed equally to the drafting process. XW, TT, XZ, JX, ZT, and CG discussed and commented on the arrangement of the manuscript. All authors were involved in the revision of the manuscript, read and approved the final manuscript version.

## Conflict of Interest

The authors declare that the research was conducted in the absence of any commercial or financial relationships that could be construed as a potential conflict of interest.
